# Tau pathology in aged cynomolgus monkeys is progressive supranuclear palsy/corticobasal degeneration- but not Alzheimer disease-like -Ultrastructural mapping of tau by EDX-

**DOI:** 10.1186/s40478-016-0385-5

**Published:** 2016-11-14

**Authors:** Toshiki Uchihara, Kentaro Endo, Hiromi Kondo, Sachi Okabayashi, Nobuhiro Shimozawa, Yasuhiro Yasutomi, Eijiro Adachi, Nobuyuki Kimura

**Affiliations:** 1 Laboratory of Structural Neuropathology, Tokyo Metropolitan Institute of Medical Science, 2-1-6 Kamikitazawa, Setagaya, Tokyo, 156-8506 Japan; 2 Histology Center, Tokyo Metropolitan Institute of Medical Science, 2-1-6 Kamikitazawa, Setagaya, Tokyo, 156-8506 Japan; 3 The Corporation for Production and Research of Laboratory Primates, Sakura 1-16-2, Tsukuba, Ibaraki 305-0003 Japan; 4 Tsukuba Primate Research Center, National Institutes of Biomedical Innovation, Health and Nutrition, Hachimandai 1-1, Tsukuba, Ibaraki 305-0843 Japan; 5 Section of Cell Biology and Pathology, Department of Alzheimer’s Disease Research, Center for Development of Advanced Medicine for Dementia, National Center for Geriatrics and Gerontology (NCGG), Moriika 7-430, Obu, Aichi 474-8511 Japan

**Keywords:** Aged monkey, Progressive supranuclear palsy, Immuno electron microscopy, Energy-dispersive X-ray analysis, Four-repeat tau

## Abstract

Concomitant deposition of amyloid -beta protein (Aβ) and neuronal tau as neurofibrillary tangles in the human brain is a hallmark of Alzheimer disease (AD). Because these deposits increase during normal aging, it has been proposed that aging brains may also undergo AD-like changes. To investigate the neuropathological changes that occur in the aging primate brain, we examined 21 brains of cynomolgus monkeys (7–36 years old) for Aβ- and tau-positive lesions. We found, 1) extensive deposition of Aβ in brains of cynomolgus monkeys over 25 years of age, 2) selective deposition of 4-repeat tau as pretangles in neurons, and as coiled body-like structures in oligodendroglia-like cells and astrocytes, 3) preferential distribution of tau in the basal ganglia and neocortex rather than the hippocampus, and 4) age-associated increases in 30–34 kDa AT8- and RD4-positive tau fragments in sarkosyl-insoluble fractions. We further labeled tau-positive structures using diaminobezidine enhanced with nickel, and visualized nickel-labeled structures by energy-dispersive X-ray (EDX) analysis of ultrathin sections. This allowed us to distinguish between nickel-labeled tau and background electron-dense structures, and we found that tau localized to 20–25 nm straight filaments in oligodendroglia-like cells and neurons. Our results indicate that the cytopathology and distribution of tau deposits in aged cynomolgus brains resemble those of progressive supranuclear palsy (PSP) and corticobasal degeneration (CBD) rather than AD. Thus, even in the presence of Aβ, age-associated deposition of tau in non-human primates likely does not occur through AD-associated mechanisms.

**Electronic supplementary material:**

The online version of this article (doi:10.1186/s40478-016-0385-5) contains supplementary material, which is available to authorized users.

## Introduction

Animal models for Alzheimer disease (AD) have been developed mainly by using genetic modifications of rodents [1]. However, these models have not been able to provide a platform to develop diagnostic and therapeutic strategies that can be transferred to clinical practice. The limitations of these models are mainly derived from their short life spans, differences in the structural organization of their brains, and molecular differences in relevant genes and their products. Furthermore, it is fundamentally unclear whether these models represent sporadic AD. In contrast, primates may provide a better model for this disease [2] based on their long life spans, the similarity of their brains to human brains, and the occurrence of age-associated deposits of amyloid beta (Aβ) and tau in their brains. Initially, only Aβ deposits were identified in these primate brains [3, 4]. However, subsequent improvements in rearing prolonged primate life span such that tau-positive lesions could also be observed [5, 6]. It is now known that primates exhibit concomitant Aβ and tau deposits, and this dual pathology has been proposed to occur through AD-like pathogenesis [7].

Tau-positive neurons in human AD brains usually exhibit immunoreactivity (IR) for both three repeat (3R) and four repeat (4R) tau in neurofibrillary tangles (NFTs) [8]. These AD-NFTs consist of tau in paired-helical filaments (PHFs), even in the early stages of the disease [9]. AD-NFTs initially develop in the parahippocampal gyrus and subsequently extend to the limbic system and then to the neocortex [10]. In contrast to AD, two other diseases with tau deposition, progressive supranuclear palsy (PSP) and corticobasal degeneration (CBD), show a distinct pathology where 4R tau is selectively deposited [11, 12], and distribution of tau lesions is not skewed towards the hippocampus. In addition, tau lesions of PSP and CBD are often found in glia as tuft-shaped astrocytes, astrocytic plaques and coiled bodies, while neuronal tau is frequently found diffusely in the cytoplasm in a pretangle state [13].

Previous studies have found abundant tau lesions in the glia of aged primate brains [6, 14, 15]. These lesions were not associated with authentic NFTs, and were found mainly in the cortex, rather than in the hippocampus, suggesting that their pathology may be distinct from that of AD. In the present study, we examined brains from cynomolgus monkeys of different ages in more detail, and found that tau-positive lesions in old animals exhibited morphological and biochemical features of PSP/CBD rather than AD.

## Materials and methods

### Brain sampling from monkeys at different ages

Twenty one brains from cynomolgus monkeys aged 7–36 years (Table 1) were obtained from the Tsukuba Primate Research Center (TPRC), National Institutes of Biomedical Innovation, Health and Nutrition (NIBIOHN), Japan. All animals were bred and maintained in an air-conditioned room at the TPRC with controlled illumination (12 h light / 12 h dark), temperature (25 ± 2 °C), humidity (60 ± 5 %), and ventilation (10 cycles/h). They were given 70 g of commercial food (CMK-2; CLEA Japan, Inc., Tokyo, Japan), and 100 g of apples daily, and unlimited access to tap water [16]. Their health status (e.g., viability, appetite, coat appearance) was assessed every morning. The maintenance of animals was conducted according to the rules for animal care of the TPRC at NIBIOHN for the care, use, and biohazard countermeasures of laboratory animals. This study was carried out in strict accordance with the recommendations in the Animal Care and Use Committee of the NIBIOHN, Japan. The protocol was approved by the Committee on the Ethics of Animal Experiments of the NIBIOHN. When a monkey presents clinical symptoms of injury or illness and was not expected to recover from pain or morbidity, it was euthanized by intravenous administration of sodium pentobarbital (>100 mg/Kg body weight). The postmortem delay between death and sampling was no more than 1 h in all animals examined. All animal experiments were conducted according to the guidelines of the Animal Care and Use Committee of National Center for Geriatrics and Gerontology (25–38) and NIBIOHN (DS17-001R5).

**Table 1:** Age and sex of the monkeys and presence or absence of AT8 or Aβ-positive lesions in their brains

Age (ys)	7	7	7	7	14	15	15	17	22	24	24	25	28	30	30	31	32	32	35	36	36
M/F	F	F	F	F	F	F	F	M	F	F	F	F	F	F	F	M	M	M		F	M
AT8	−	−	−	−	−	−	−	−	−	−	−	−	−	−	+	+	−	+	NA	+	++
Aβ	−	−	−	−	−	−	−	−	−	−	+	+	−	+	+	−	+	+	NA	+	++
WB	*	*	*	*													*	*	*	NA	*

*Abbreviations: Age* age at death, +presence of lesions, −absence of lesions, *M* male, *F* female, *NA* not available, *WB* Western blot

*samples used for WB

### Immunohistochemistry

Brain samples were immersion fixed in 4 % paraformaldehyde (PFA), cut into serial coronal slices from frontal to occipital lobes and embedded in paraffin. Five μm-thick sections were deparaffinized and subjected to appropriate pretreatments and immunohistochemistry with the antibodies listed in Table 2 [11, 12]. Epitopes of these antibodies (AT8 [17, 18], RD3 for 3R tau and RD4 for 4R tau [11]), necessary pretreatments, and specificity for RD3 and RD4 have been described previously as summarized in Table 2 [12]. Briefly, deparaffinized sections for RD3 and RD4 immunostains were treated with potassium permanganate for 15 min followed by 2 % oxalic acid for 3 min, 100 % formic acid for 30 min each at room temperature and then heat retrieved with 0.01 M citrate buffer in pressure cooker (115 °C for 10 min). Samples were then incubated in primary antibodies diluted with phosphate-buffered physiological saline (PBS) at 4 °C for 2 days, and biotinylated secondary antibodies (1:1000, Vector, Burlingame, CA) for 2 h. After subsequent incubation with streptavidin biotinylated horseradish peroxidase complex (ABC Elite, Vector, Burlingame, CA), color development was performed with diaminobenzidine (DAB) in the presence of imidazole and nickel ammonium chloride. For double immunohistochemistry for PHF-tau (AT8) and Aβ42, sections were initially incubated with AT8, and visualized with DAB containing nickel ammonium sulfate, which generates dark purple precipitates. The same section was then incubated with an anti-Aβ42 antibody, and visualized with DAB, which generates brown precipitates.

**Table 2:** List of antibodies used in this study

Antibody (clone)	Epitope	Species	Dilution	Pretreatment	Supplier
PHF-tau (AT8)	pS202/pT205(/pS208) [17, 18]	mo	1:10,000	none	Thermo Fisher, Waltham, MA
RD3 (8E6/C11)	KHQPGGGKVQIVYKPV [11]	mo	1:3000	KMn, Ox, FA, AC [12]	Upstate, Lake Placid, NY
RD4 (1E1/A6)	VQIINKKLDLSNVQSKC [11]	mo	1:1000	KMn, Ox, FA, AC [12]	Upstate, Lake Placid, NY
Aβ42		rb	1:20,000	FA	IBL, Fujioka, Japan

*Abbreviations*: *mo* mouse, *KMn* 0.25 % potassium permanganate for 15 min, *Ox* 2%oxalic acid for 3 min, *FA* 99%formic acid for 30 min, *AC* autoclaving in 0.01 M citrate buffer at 121 °C for 30 min, *rb* rabbit

In order to plot AT8-positive lesions, the entire area of immunostained-sections was digitalized with a virtual slide system (VS120, Olympus, Tokyo, Japan). Obtained images were displayed on Canvas 12 (ACD Systems of America, Seattle, WA) and each AT8-positive lesion was classified into neuronal (red ring), astrocytic (green ring) and oligodendroglia-like (blue spot). Plotting was performed manually on transparent layers, overlaid on the original virtual slide images (×10 objective). When captured images were ambiguous, the nature of each lesion was assessed from the original slide using a microscope. Silver impregnation was performed using Gallyas and Campbell-Switzer methods on slices adjacent to those used for immunohistochemistry [19].

### Immunoelectron microscopy with diaminobenzidine

A PFA-fixed specimen from the temporal lobe of the 36 year-old monkey was sliced using a sliding microtome. Slices were treated with 0.5 % hydrogen peroxide for 1 h, and then incubated with AT8 antibody (1:1000) for more than 10 days at 4 °C. Immunoreactions were developed using the avidin biotin-peroxidase method described above. After washing in 0.1 M phosphate buffer (PB, pH 7.4), slices were fixed with 2.5 % glutaraldehyde in PB and postfixed with 2 % osmium tetroxide in PB for 2 h. They were dehydrated in a graded series of ethanol concentrations followed by propylene oxide, and then horizontally embedded in epoxy resin (EPON 812, TAAB, Aldermaston, UK). Embedded samples were sectioned into 4 μm-thick serial semi-thin sections. Lesions were identified based on the DAB-labeled products under a light microscope. Sections containing target lesions were adhered on another block of epoxy resin. Ultrathin sections were cut, stained with uranyl acetate and lead citrate, and examined under a transmission electron microscope (TEM, JEM-1400, JEOL, Tokyo, Japan) or a scanning transmission electron microscope (STEM, Hitachi HD-2700, Hitachi High Technologies Corporation, Tokyo, Japan). The STEM was equipped with a cold-field emission gun and detectors that consist of bright-field, high-angle annular dark-field and secondary electron detectors to distinguish different elements (including nickel, osmium, lead, uranium) based on their energy spectra. This was used to identify the presence of these elements in each STEM pixel in the entire EM field to map the distribution of each element in relation to underlying ultrastructures [9, 20].

### Biochemical analyses of monkey brains

The preparation of sarkosyl-insoluble fractions has been previously described [21]. Briefly, frozen temporal cortices (0.2 g) were homogenized in a glass homogenizer in 4 ml of TBS (10 mM Tris, 150 mM NaCl, pH 7.4) containing Complete Mini TM proteinase inhibitor cocktail (Roche Molecular Biochemicals, Penzberg, Germany) and phosphatase inhibitors (1 mM NaF, 0.4 μM Na_3_VO_4_, and 0.5 μM okadaic acid). After centrifugation at 24,000 g for 15 min, the supernatant was collected as the TBS-soluble fraction. Sarkosyl-insoluble, PHF-enriched fractions were prepared from the TBS-insoluble precipitates. Precipitates were re-homogenized in 4 ml of TBS containing 0.32 M sucrose and centrifuged at 24,000 g for 15 min. One tenth volume of 10 % sarkosyl solution was added to supernatants, which were then vortexed, incubated for 1 h at 37 °C, and centrifuged at 150,000 g for 1 h. Resulting pellets were washed in TBS and centrifuged at 150,000 g for 1 h to obtain the sarkosyl-insoluble fractions (pellets). 10 μg of proteins from TBS-soluble and the sarkosyl-insoluble fractions were then subjected to SDS-PAGE, and blotted onto polyvinylidene fluoride membranes. Membranes were blocked with 3 % bovine serum albumin in PBS (pH 7.0) containing 0.1 % Tween-20 (PBST) (for phosphorylated-tau) or 5 % nonfat dried milk in PBST (for other proteins) for 1 h at room temperature, and then incubated with primary antibodies, AT8 (1:500), RD3 (1:1000), or RD4 (1:1000), overnight at 4 °C. Membranes were then incubated with horseradish peroxidase-conjugated goat anti-mouse IgG (Cell Signaling Technology, Danvers, MA) for 1 h at room temperature. Immunoreactive elements were visualized using enhanced chemiluminescence (Immobilon Western Detection Reagents; Millipore, Billerica, MA).

## Results

**Fig. 1:**
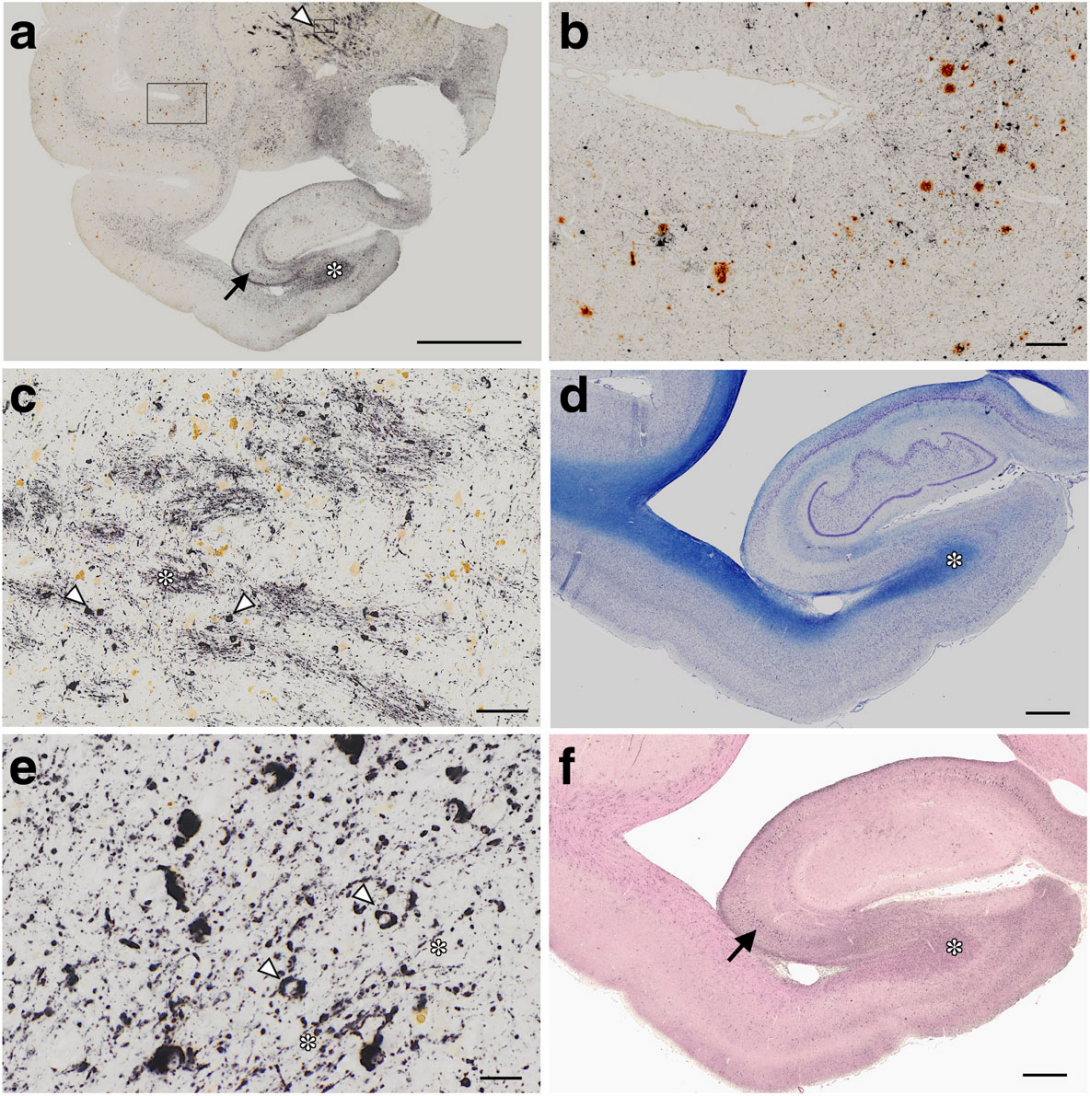
Amyloid beta and tau deposition in the 36-year-old monkey. Double staining for PHF tau (AT8, dark purple or black) and Aβ (brown) in brain slices from the 36-year-old monkey **a**. Pyramidal neurons were positive for AT8 (arrow, a). Higher magnification of a temporal lobe region, indicated by the large rectangle in **a**, is shown in **b**, again showing AT8-positive neurons in dark purple, and Aβ-positive deposits in brown. AT8 immunoreactivity (IR) was abundant in the globus pallidus (arrowhead in a) and in the white matter of the hippocampus (asterisk in **a** and **d**). In higher magnification images of the globus pallidus **c**, AT8 IR was found along fiber bundles (asterisk) and small round cells (arrowheads. See Fig. 4 for more details). Brown deposits are macrophages containing hemosiderin. Higher magnification of the hippocampal white matter (asterisk **d** and **e**) revealed AT8-positive oligodendroglia-like cells (arrowheads in **e**) and fibers (asterisks in **e**). The hippocampal white matter (asterisk, **f**) and pyramidal neurons (arrow, **f**) were also immunopositive with RD4 (dark purple staining in **f**) but not for RD3 (data not shown). Images (**a**–**f**) were captured using a virtual slide system VS120 (Olympus, Tokyo, Japan), equipped with “extended focus imaging”. Multiple images at different focal planes (17 planes with an interval of 0.5 μm) were fused into a single image. **a**–**c**, **e**: double staining for AT8 (dark purple) and Aβ (brown); **d**: Klüver-Barrera stain; **f**: RD4 immunostaining counter stained with nuclear fast red. Bars **a**:5 mm; **b**:200 μm, **c**:100 μm; **d**, f:1 mm, **e**:20 μm

In this cohort, deposits immunoreactive for Aβ were found in brain sections from primates 24 years of age and older, while deposits immunoreactive for PHF-tau (AT8) were found starting at 30 years of age (Table 1). The brain from the oldest monkey we examined (36 yo) exhibited numerous tau deposits (dark purple, Fig. 1a, b) as well as Aβ deposits (brown, Fig. 1a, b). Pyramidal neurons in the hippocampus exhibited AT8 IR (Fig. 1a, arrow). Because tau-positive deposits were abundant in the white matter (Fig. 1a, d, asterisks) and basal ganglia, (Fig. 1a, arrowhead) particularly in the globus pallidus (GP, c), their distribution was different from that of AD in the human brain, which is prevalent in the hippocampus.

**Fig. 2:**
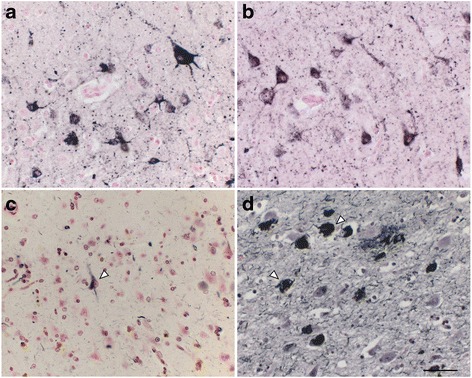
Pretangle neurons in the hippocampal pyramidal layer of the 36-year-old monkey. Tau IR with AT8 **a** and that with RD4 **b** were granular and diffuse in the neuronal cytoplasm and dendrites, rarely organized into neurofibrillary tangles. Argyrophilia with Gallyas silver impregnation was limited to a few neurons (**c**, arrowhead). None of these neurons exhibited fibrillary structures in their cytoplasm. In contrast, numerous neurofibrillary tangles were stained by Gallyas silver impregnation in the CA1 of a 94-year-old male with AD (**d**, arrowheads). Scale bar, 50 μm

In the GP, AT8 immunoreactivity (IR) was found in fiber bundles (asterisk, c) and small round cells, likely to be oligodendroglia (arrowheads, c. See Fig. 4 for more details). AT8 IR was also abundant in the white matter in the temporal lobe (a, asterisk) and the hippocampus (asterisks in a and d). Higher magnification of the hippocampal white matter (e) identified AT8-positive oligodendroglia-like cells (arrowheads, e) and fibers (asterisks, e). Adjacent hippocampal slices demonstrated 4R tau (RD4, Fig. 1f) IR in pyramidal neurons (arrow, Fig. 1f) and in the hippocampal white matter (asterisk, Fig. 1f). Because 3R tau IR was absent in these AT8/RD4-positive lesions (data not shown), their staining profile was different from that of human AD, which is immunopositive for both 3R and 4R tau [11, 12]. At higher magnifications, AT8 (Fig. 2a) and RD4 (Fig. 2b) tau immunoreactivity was diffuse and granular in the neuronal cytoplasm and dendrites, and rarely organized into NFTs. Argyrophilia with Gallyas silver impregnation was quite limited (Fig. 2c, arrowhead) and they were not argyrophilic with Campbell-Switzer silver impregnation (data not shown). The scarcity of NTFs, fibrillary structure, and Gallyas silver argyrophilia, also differs from AD pathology in the human brain (Fig. 2d, arrowheads).

**Fig. 3:**
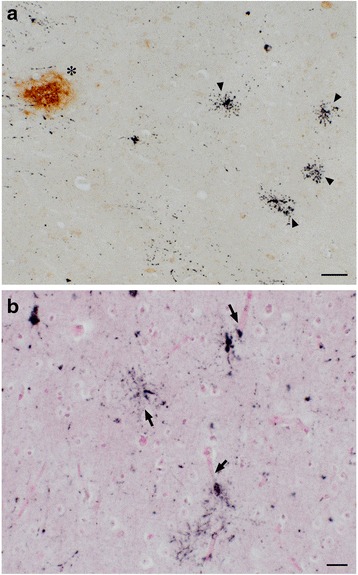
Tau-positive astrocytes around blood vessels. AT8-positive astrocytes (dark purple staining, indicated by arrowheads) were found independently of Aβ deposits (brown staining, indicated by asterisk) in the temporal cortex **a**. RD4-positive signals (purple staining), but not for RD3 (data not shown), were found close to blood vessels (arrows) in the same region **b**. Scale bars, a:50 μm, b:20 μm

**Fig. 4:**
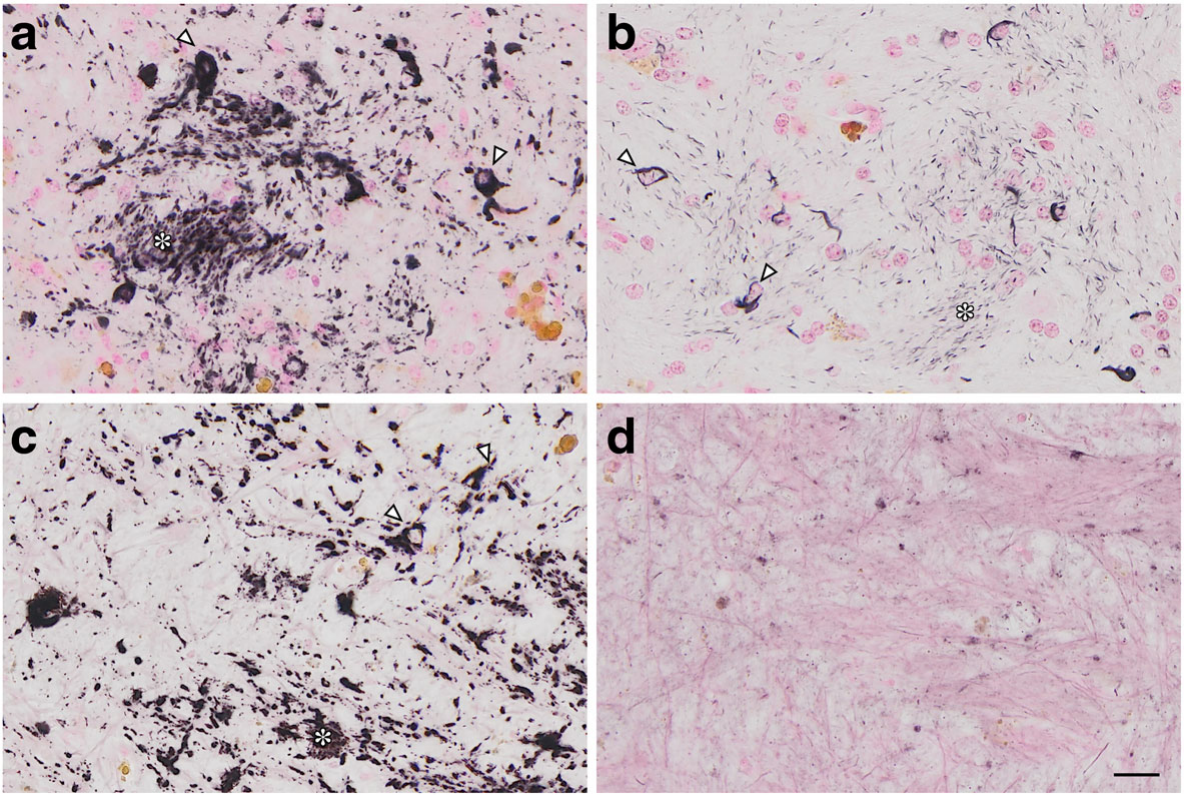
Tau-positive oligodendroglia-like cells and threads in the globus pallidus. In the globus pallidus of the 36 yo monkey, small oligodendroglia-like cells (arrowheads) and threads (asterisks) were positive with AT8 **a**. Argyrophilia with Gallyas silver impregnation was also observed **b**. Adjacent slices were positive with RD4 **c** but negative with RD3 **d**. Extended focus imaging (**a**–**d**, see legend of Fig. 1), Scale bar, 20 μm

We observed numerous AT8-positive processes (Fig. 3a, dark purple, arrowheads), independent of Aβ (Fig. 3a, brown, asterisk) in the temporal cortex. Similar structures in the same area were positive exclusively for RD4 (Fig. 3b) and oriented around blood vessels (Fig. 3b, arrows). Thorn-shaped astrocytes or granular/fuzzy astrocytes grouped under the umbrella of “aging-related tau astrogliopathy” were not apparent in these monkey brains [22]. AT8-positive oligodendroglia-like cells and threads were abundant in GP (Fig. 4a) and in the white matter (Fig. 1e). Both perikarya and threads of these oligodendroglia-like cells were positive for both Gallyas silver impregnation (Fig. 4b), and RD4 (Fig. 4c) in adjacent sections, but consistently negative for 3R (Fig. 4d). In summary, AT8/RD4-positive neurons were pretangle-like but their distribution was accentuated in pyramidal neurons of Ammon’s horn (Fig. 1a and f arrow) as in AD. However, AT8/RD4-positive glial cells in the hippocampal white matter (Fig. 1a, d and f asterisks) and basal ganglia were conspicuous.

**Fig. 5:**
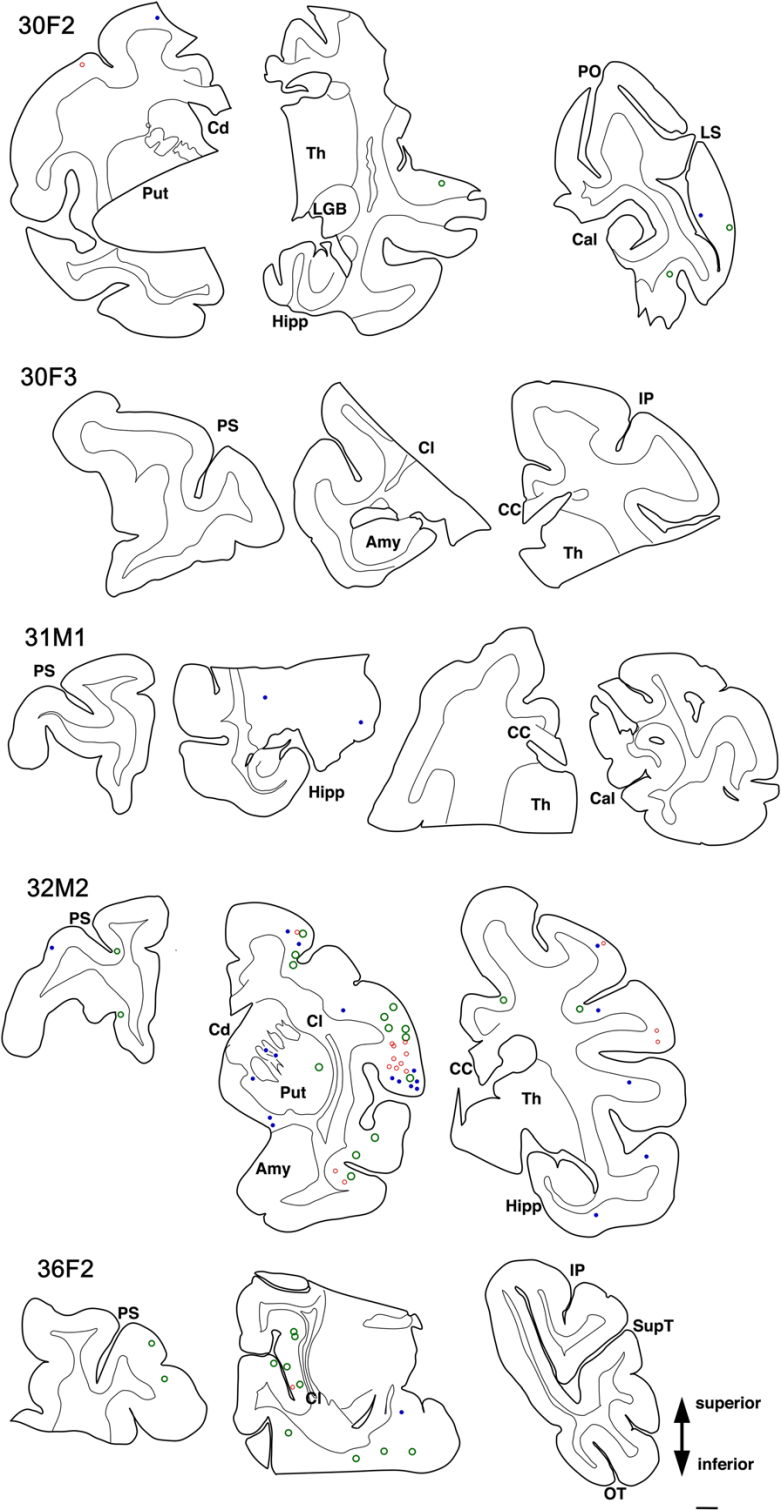
Frontal-dominant distribution of tau-positive neurons and glia. AT8-positive structures were plotted onto coronal slices with astrocytic signals labeled as green rings, neuronal signals labeled as red rings and signals in oligodendroglial cells labeled as blue spots. Slices are displayed from the left (anterior) to the right (posterior), with Case IDs indicated for each row (eg, “30 F2” indicates “30-year-old female, case no 2”). AT8-positive lesions were detectable after age 30, but these were few in number. These lesions were predominantly in the neocortex rather than in the hippocampus. Amy:amygdala; Cal:calcarine sulcus; CC:corpus callosum; Cd:caudate nucleus; Cl:claustrum; Hipp:hippocampus; IP:intraparietal sulcus; LGB:lateral geniculate body; LS:lunate sulcus; PO:parietooccipital sulcus; PS:principal sulcus; Put:putamen; SupT:superior temporal sulcus; OT:occipitotemporal sulcus; Th:thalamus; Scale bar, 2 mm

Tau-positive lesions were particularly numerous in the oldest monkey examined (36 yo Fig. 1). However, these cytopathological findings, including pretangle forms of tau in neurons, tau-positive astrocytes, and inclusions in oligodendroglia-like cells, were also detected in brain sections from other monkeys older than 30 years of age (Fig. 5). Similar to the 36-year old monkey, tau-positive lesions were seen more frequently in the neocortex than in the hippocampus (Fig. 5).

**Fig. 6:**
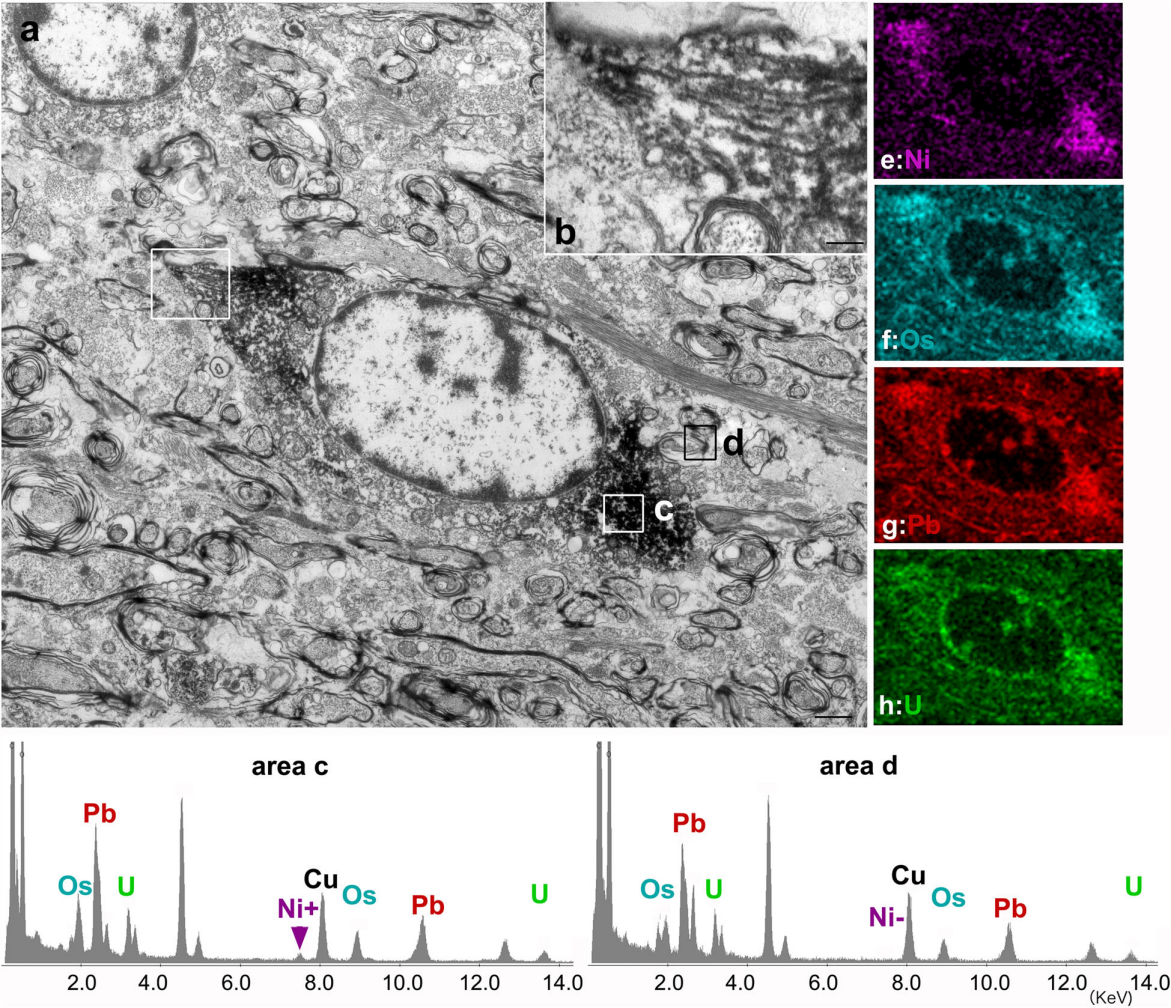
Immunoelectron microscopy of tau-positive oligodendroglia-like cells. Oligodendroglia-like cells **a** labeled with AT8/diaminobenzidine (DAB, Scale bar, 1 μm). Nickel (Ni) ammonium sulfate was used to enhance the DAB signals. A higher magnification image of the white box in the upper left of **a** is shown in **b** and shows AT8 labelled 20–25 nm diameter straight filaments (scale bar:0.2 μm). The specificity of this labeling was confirmed by energy-dispersive X-ray (EDX) analysis. A nickel peak (Ni+) was detected in areas with DAB precipitates (area **c**) but not in background areas (Ni-, area **d**). EDX mapping of this Ni peak identified areas labeled specifically by DAB (**e**, Ni, purple). In contrast, EDX mapping for osmium (**f**, Os, cyan), lead (**g**, Pb, red) and uranium (**h**, U, green), identified background electron-dense areas. For a high resolution image of Fig. 6 please see Additional file 1

Oligodendroglia-like cells in the hippocampal white matter were identified on AT8/DAB stained sections embedded in Epon and trimmed for electron microscopic examination (Fig. 6a). In these cells, non-twisted filaments with a diameter of 20–25 nm were randomly scattered with occasional parallel streams in the cytoplasm (Fig. 6b, inset). Because ultrastructures and DAB labeling are both electron-dense, distinguishing between DAB labeled structures and ultrastructures is difficult. To address this problem, we first immunolabeled with DAB in the presence of ammonium nickel sulfate, so that DAB precipitates contained nickel. We next analyzed sections using STEM and energy dispersive X-ray (EDX) analysis to identify Ni-specific energy peaks (Fig. 6a, compare area c, Ni+, with area d, Ni-). By plotting the presence or absence of Ni in each pixel in the EM field, we were able to accurately map tau IR. We further show that the distribution of Ni (Fig. 6e Ni, purple) is distinct from the distribution of components of non-specific electron dense regions, including osmium (Fig. 6f Os, cyan), lead (Fig. 6g Pb, red) or uranium (Fig. 6h U, green).

**Fig. 7:**
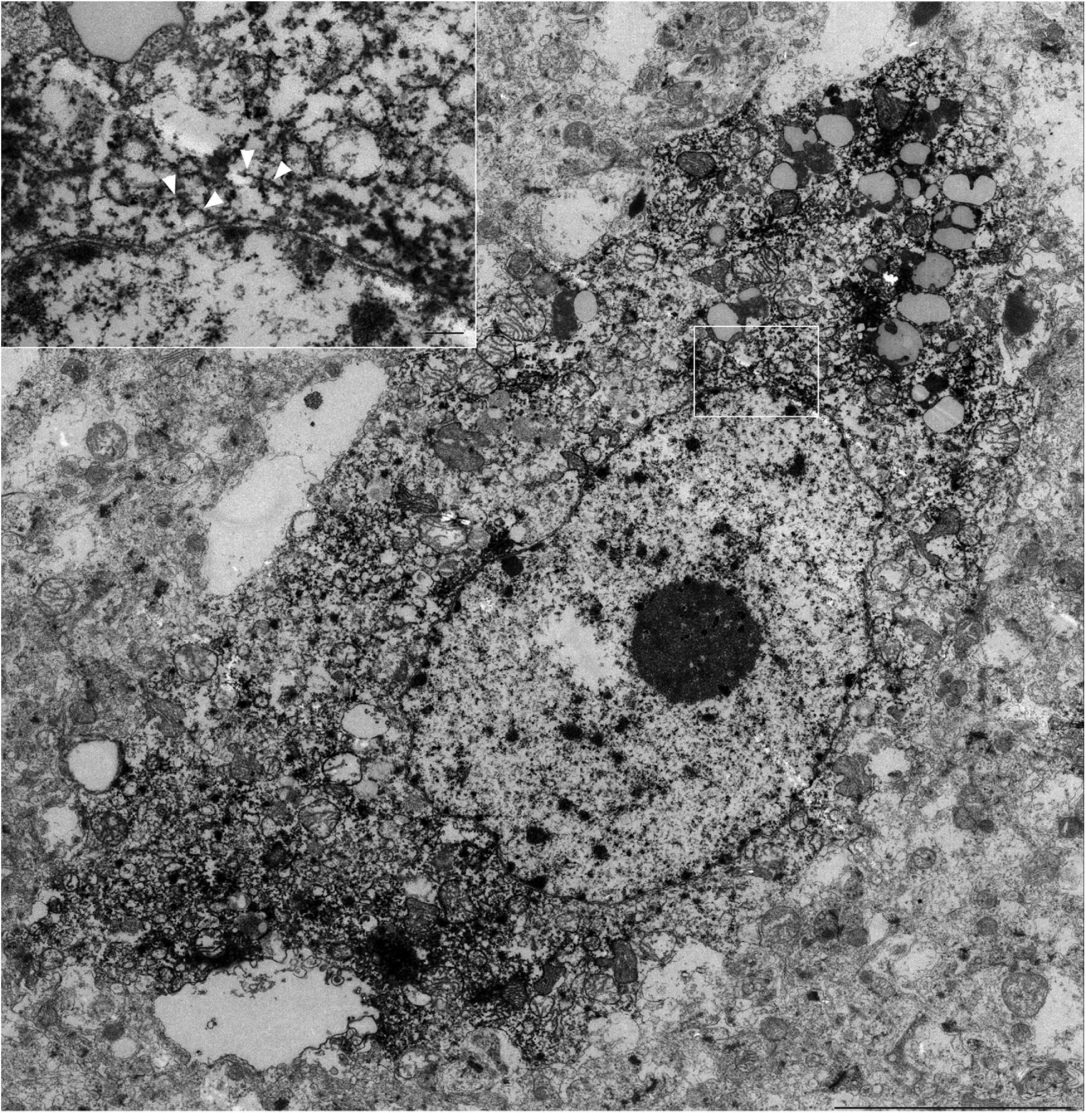
Immunoelectron microscopy of a neuron containing tau-positive signals. Tau immunolabeled filaments (arrowheads) were observed around the nuclear membrane (inset, bar:0.2 μm), although tau-positive deposits were not abundant in neurons (scale bar, 5 μm). For a high resolution image of Fig. 7 please see Additional file 2

When we analyzed neurons using this technique, we found similar electron-dense labeling in the neuronal cytoplasm, sometimes in close contact with the outer nuclear membrane (Fig. 7). Solid filamentous structures, as seen in oligodendroglia-like cells, were present, but rare in neurons (Fig. 7 inset, arrowheads). The scarcity of filamentous structures in neurons may be correlated with less intense argyrophilia on Gallyas silver impregnation (Fig. 2c).

**Fig. 8:**
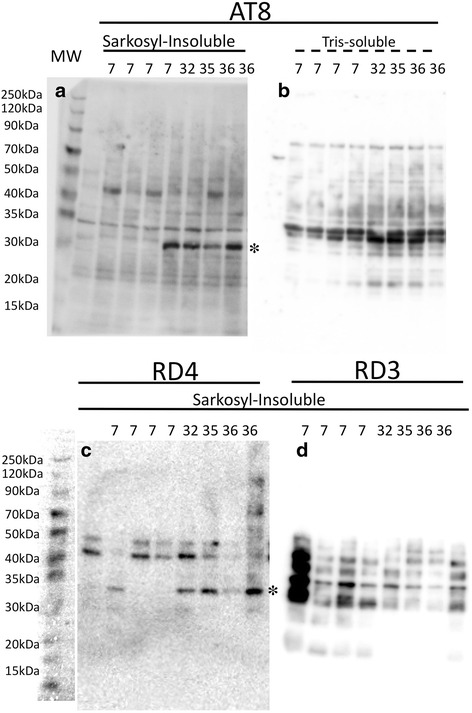
Temporal lobe homogenates probed with AT8 and isoform-specific antibodies, RD3 and RD4. In sarkosyl-insoluble preparations, AT8-positive **a** and RD4-positive **c** bands were detectable between 30 and 35KDa in aged monkeys (asterisk 32, 35, 36 and 36 years-old) but not in young controls (7 years-old). This age associated increase is neither evident in Tris-soluble preparations probed by AT8 **b**, nor in sarkosyl-insoluble preparations probed with RD3 **d**. Higher molecular complex at 50–70 kDa, seen in human brain with tau deposits, were not detectable in either sarkosyl-insoluble preparations or tris-soluble preparations

To characterize age-associated changes in tau protein biochemically, we next separated extracts from frozen temporal lobes of young (7 yo), and old (over 30 yo) monkeys into TBS-soluble and sarkosyl-insoluble fractions and probed for AT8-positive (Fig. 8a) and RD4-positive (Fig. 8c) bands by western blotting. In sarkosyl-insoluble fractions, we identified AT8 or RD4-positive bands between 30 and 35KDa in aged monkeys (asterisks) but not in young controls (7 yo). These bands were not found in TBS-soluble fractions (Fig. 8b), or in sarkosyl-insoluble fractions probed with RD3 antibodies (Fig. 8d). Surprisingly, higher molecular complexes at 50–70 kDa, seen in human brains with tau deposits [23, 24], were neither detectable in the sarkosyl-insoluble preparations nor in the TBS-soluble preparations. These findings were confirmed in duplicate experiments.

## Discussion

### Aβ and tau-positive deposits in aged monkey brains

Concomitant age-dependent accumulation of Aβ and neuronal tau deposits in the brains of primates [4, 6] has led to the proposal that AD-like pathology may occur during normal aging in primates. However, it has been unclear whether these deposits are really representative of AD pathogenesis or not.

### PSP-like cytopathological alterations in aged monkey brains

In all brains examined in this study, tau-positive structures exhibited immunoreactivity for 4R, but not 3R tau. Tau-positive structures were also argyrophilic with Gallyas, but not with Campbell-Switzer silver impregnation. This cytopathology is different from that of AD, which usually exhibits immunoreactivity for both 3R and 4R tau, and argyrophilia with both Gallyas and Campbell-Switzer silver impregnations [19]. Instead, these phenotypes suggest that a PSP or CBD-like cytopathology may occur in brains of aged primates. Supporting this idea, the 4R-positive structures that we identified around blood vessel in old primates (Fig. 3b) are similar to tau-positive structures identified in tuft-shaped astrocytes in PSP and astrocytic plaques in CBD [25]. Further, we did not observe tau IR in neurites around senile plaques (Fig. 3a, asterisk), suggesting that most tau-positive structures in old primate brains may be of glial origin similar to human PSP/CBD, which are not frequent in aged human brains with AD pathologies. Moreover, these tau-positive astrocytes are morphologically different from thorn-shaped astrocytes, granular/fuzzy astrocytes grouped under the umbrella of “aging-related tau astrogliopathy [22]” Consistent with our results, Kiatipattanasakul and colleagues [26] previously described PSP-like tau deposition in neurons and glia in an aged (35 yo) albino cynomolgus monkey. They also observed Gallyas-positive glia in the basal ganglia, thalamus, brainstem and the white matter as well as NFTs in the thalamus. However, they proposed that the occurrence of PSP-like cytopathology in their monkey brain was exceptional. In our study, however, we observed similar neuronal and glial cytopathological alterations of tau in 5 out of 7 monkeys over 30 years of age (Table 2, Fig. 5), suggesting that PSP-like cytopathologies may instead represent a common aspect of aged monkey brains.

### PSP-like distribution of tau cytopathologies in aged monkey brains

Although tau-positive lesions were found in temporal and hippocampal areas in the oldest monkey examined (36 M, Fig. 1), lesions were also abundant in the white matter and basal ganglia, predominantly in oligodendroglia-like cells and as intrafascicular threads. In other monkeys over 30 years of age, lesions were present in the frontal and temporal neocortices and basal ganglia rather than in the hippocampus (Fig. 5). Again, this distribution of tau-positive lesions is reminiscent of the aged cynomolgus monkey described by Kiatipattanasakul et al. [26], and suggest cytopathological alterations characteristic of PSP rather than those of AD [10, 13]. A similar cortical distribution of tau lesions, different from human AD, has been reported in the brains of gorilla [14, 15]. Taken together, tau-positive lesions in aged non-human primate brains do not necessarily represent AD-like pathogenesis even though Aβ deposits may be present in the same specimens.

### EDX isolation of DAB-Ni labeling from the background

In our previous studies [20], we distinguished between electron-dense areas due to immunolabeling from background electron-dense areas by labeling with Quantum dot nanocrystal (QD). This EDX detection of the QD constituents, selenium and cadmium, was further extended to map these elements by highlighting against background structures (EDX mapping) [9]. In our current study, we developed a novel technique of immunoelectron microscopy (immuno EM) by applying this EDX mapping on ultrathin sections, developed with DAB enhanced with nickel ammonium sulfate. Because EDX spectra can detect the presence of nickel in DAB decorated areas (Fig. 6a, area c, Ni+), and because nickel is not found in background areas (Fig. 6a, area d, Ni-), it was possible to map pixels containing nickel over the entire EDX field (Fig. 6e, Ni, violet) to identify specific DAB-positive regions. Similar maps for peaks of osmium (Fig. 6f, Os, cyan), lead (Fig. 6g, Pb, red) and uranium (Fig. 6h, U, green) identified background electron-dense regions that were qualitatively different from the Ni-specific maps. This approach, easily performed by analyzing DAB-Ni decorated sections with EDX, may greatly expand the utility and reliability of DAB-Ni decorated EM preparations by distinguishing specific signals from otherwise indistinguishable backgrounds of similar electron density.

### PSP-like ultrastructure of tau filaments in oligodendroglia-like cells

4R tau labeling and Gallyas argyrophilia were more intense in oligodendroglia-like cells in the white matter (Fig. 4b) than in neurons (Fig. 2c). In these oligodendroglia-like cells, AT8-labeled fibrils were 20–25 nm in diameter without apparent constriction (Fig. 6b), similar to those reported in tau-positive fibrils in oligodendroglia in PSP brains [27]. In contrast, in oligodendrotcytes in AD brains, mixtures of straight filaments with a diameter of 16 nm, and irregularly constricted filaments with greatest width of 30 nm have been described [28].

In our study, neurons in aged monkeys were positive, specifically for 4R tau, but argyrophilia with Gallyas was much less intense, suggesting an early, premature state of tau deposition, and aggregation as pretangle neurons. It was possible to identify some AT8-decorated fibrils in the neuronal cytoplasm (Fig. 7 inset, arrowheads), but these were much less abundant than in oligodendrocytes, and their random arrangement without forming PHFs resembles pretangle neurons in CBD [9].

### PSP-like tau fragments in aged monkey brains

The sarkosyl-insoluble fraction from aged monkey brains (32–36 yo) contained 30–34 kDa bands positive for AT8 and RD4, not found in young controls (7 yo) (Table 1, Fig. 8). Tau-positive bands in this molecular range have been reported to be specific to PSP, and are distinct from AD [23, 24]. Curiously, full-length hyperphosphorylated tau (60, 64 and 68 kDa, Fig. 8a, c) was not detected in sarkosyl-insoluble fractions from young or aged monkeys, and it remains to be clarified how these shorter tau fragments are generated. The absence of full-length tau in these fractions may be related to scarcity of neuronal tau (Fig. 7), while solid fibrillary structures in oligodendroglia-like cells (Fig. 6) may correspond to the 30–34 kDa sarkosyl-insoluble fragments. Thus, it is possible that neuronal tau in human brains with PSP consists mainly to full-length tau, while glial tau consists of the 30–34 kDa fragments, although this interpretation remains to be proved.

### Is the pathology of aged cynomolgus brains AD-like or PSP-like?

Preferential limbic-hippocampal accumulation of tau-positive neurons, similar to human AD pathology, has been described in aged baboons [29], captive cheetahs (*Acinonyx jubatus*) [7], wild Tsushima leopard cats [30], and domestic cats [31]. Moreover, aged domestic cats’ brains exhibit both 3R and 4R tau immunoreactivity in sarkosyl-insoluble brain fractions [31], again consistent with characteristics of AD. However, in this study, we found that tau lesions in aged monkey brains are similar to lesions of PSP, and distinct from those of AD, even in the presence of coexisting Aβ deposits (Table 1). Consistent with our results, Rosen and colleagues [32] identified tau lesions not associated with Aβ plaques in a 41 yo chimpanzee. These lesions were most abundant in the prefrontal cortex, with decreasing amounts in the temporal and occipital cortices, a distribution distinct from that of AD, which typically shows a hierarchical distribution around the hippocampus [10]. Tau lesions were also found in the GP and neostriatum in this chimpanzee, suggesting a PSP-like origin for these lesions, despite the presence of Aβ. Similar neocortical predominance of tau lesions has been reported in *Microcebus murinus* [33] and gorillas [14, 15]. Further, Oikawa and colleagues identified tau-positive neurons and glia, with less evident argyrophilia with Gallyas silver impregnation [6]. This result is consistent with repeated observations that true NFTs are extremely rare in nonhuman primate brains [34, 35], and suggests a premature state of tau deposition as “pretangle neurons” in aged cynomolgus monkeys. Finally, widespread development of tau/Gallyas-positive neurons and glia (tufts of abnormal fibers, thorn-shaped astrocytes, glial coiled bodies and argyrophilic threads) in the basal ganglia and brainstem nuclei have been reported in a 35 yo albino cynomolgus monkey [26]. Even with coexisting Aβ deposits, these tau-positive structures are more consistent with a PSP-like rather than AD-like pathology.

## Conclusions

It is generally accepted that the co-existence of Aβ deposits and tau-positive lesions provides a firm histological basis for the diagnosis of AD. However, close scrutiny of tau-positive structures in our cohort of cynomolgus monkeys demonstrated a constellation of pathological findings, such as pretangle neurons and tau-positive glia (oligodendrocyte-like cells and astrocytes) prevalent in the neocortex and basal ganglia, which may favor the histological diagnosis of PSP rather than AD. Indeed, some of these PSP-like features of tau have been described previously in animal brains. In human brains, as well, Aβ deposits have been described in PSP brains [36, 37]. In human PSP, tau-positive lesions seem to occur independently of Aβ deposits, and it is unlikely that Aβ deposition induces PSP-like tau pathology even if the former precedes the latter. Our results suggest that it is necessary to recognize how Aβ and tau are represented in animal brains without being preoccupied by AD-pathology models.

## Additional files

Additional file 1: Immunoelectron microscopy of tau-positive oligodendroglia-like cells. (JPG 7.94 mb)
Additional file 2: Immunoelectron microscopy of a neuron containing tau-positive signals. (JPG 8.57 mb)

## Abbreviations

3R: Three repeat
4R: Four repeat
AD: Alzheimer disease
Aβ: Amyloid-beta protein
CBD: Corticobasal degeneration
DAB: Diaminebenzidine
EDX Analysis: Energy-dispersive X-ray analysis
EM: Electron microscopy
GP: Globus pallidus
IR: Immunoreactivity, Ni, nickel
PHF: Paired helical filament
PSP: Progressive supranuclear palsy
QD: Quantum dot
STEM: Scanning transmission electron microscopy
TEM: Transmission electron microscopy
