# Supplementary figures and images for: Tau pathology in aged cynomolgus monkeys is progressive supranuclear palsy/corticobasal degeneration- but not Alzheimer disease-like -Ultrastructural mapping of tau by EDX-
Source: Acta Neuropathol Commun. 2016 Nov 14;4:118. doi: 10.1186/s40478-016-0385-5 (PMC5109723; doi:10.1186/s40478-016-0385-5)

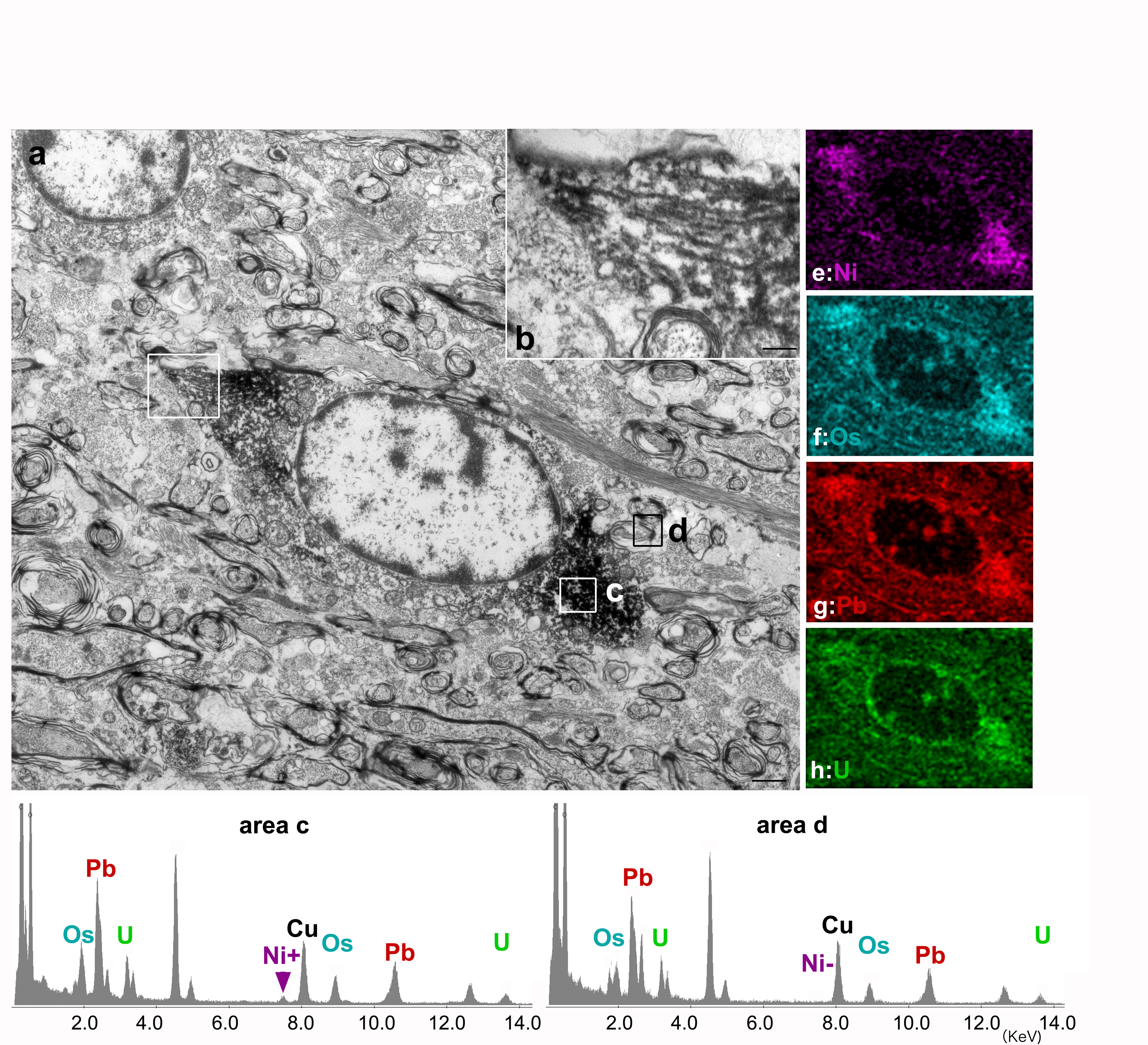

Supplement: Additional file 1: — Immunoelectron microscopy of tau-positive oligodendroglia-like cells. (JPG 7.94 mb) [file 40478_2016_385_MOESM1_ESM.jpg]

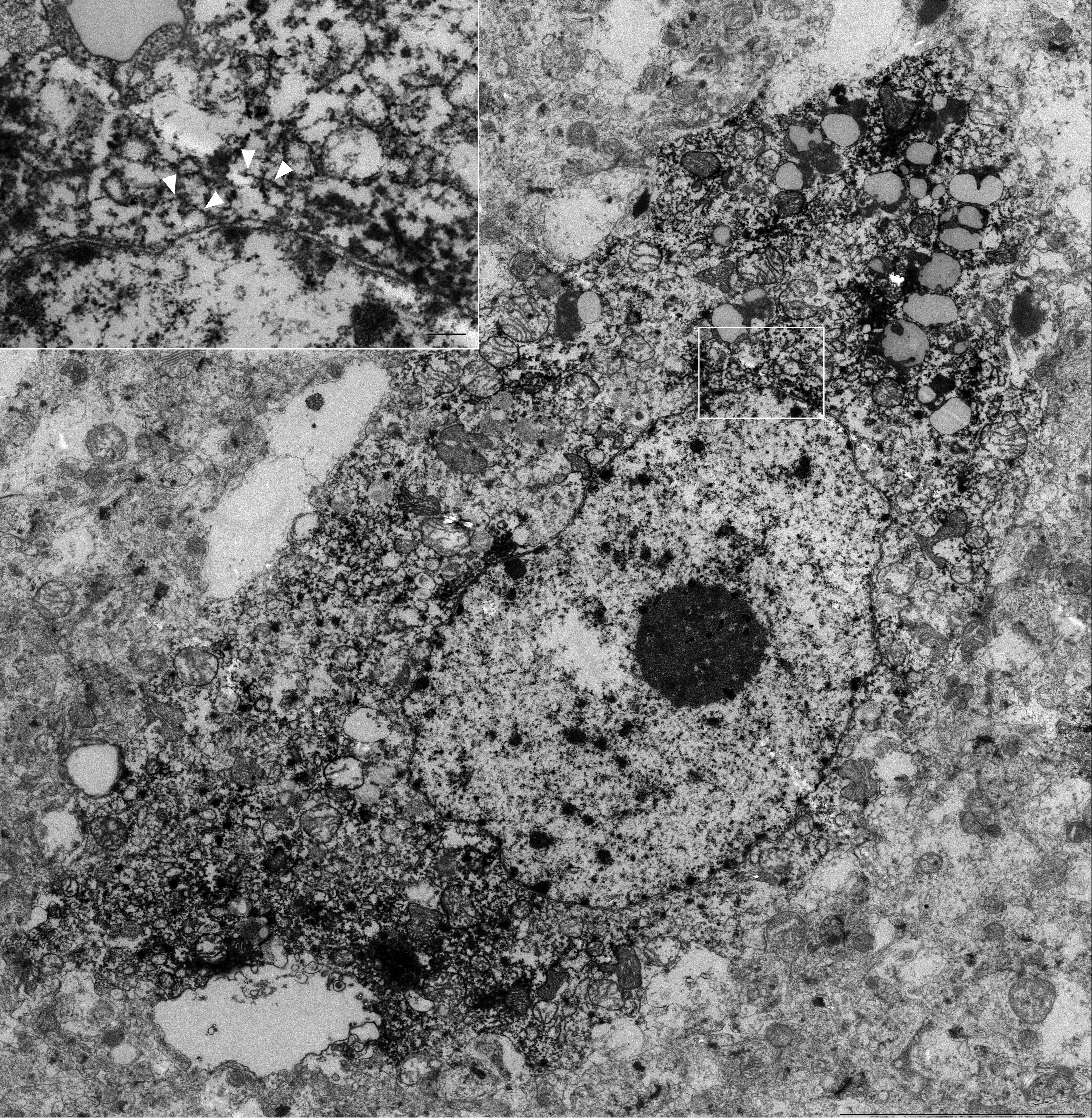

Supplement: Additional file 2: — Immunoelectron microscopy of a neuron containing tau-positive signals. (JPG 8.57 mb) [file 40478_2016_385_MOESM2_ESM.jpg]
